# Small Moving Vehicle Detection in a Satellite Video of an Urban Area

**DOI:** 10.3390/s16091528

**Published:** 2016-09-21

**Authors:** Tao Yang, Xiwen Wang, Bowei Yao, Jing Li, Yanning Zhang, Zhannan He, Wencheng Duan

**Affiliations:** 1 ShaanXi Provincial Key Lab of Speech and Image Infromation Processing, School of Computer Science, Northwestern Polytechnical University, Xi’an 710129, China; watermelonwxw121@163.com (X.W.); ybwfairy@163.com (B.Y.); ynzhangnwpu@gmail.com (Y.Z.); zhannanhe@mail.nwpu.edu.cn (Z.H.); duanwch@mail.nwpu.edu.cn (W.D.); 2 School of Telecommunications Engineering, Xidian University, Xi’an 710071, China; jinglixd@mail.xidian.edu.cn

**Keywords:** moving vehicle detection, satellite video, motion heat map, local saliency map

## Abstract

Vehicle surveillance of a wide area allows us to learn much about the daily activities and traffic information. With the rapid development of remote sensing, satellite video has become an important data source for vehicle detection, which provides a broader field of surveillance. The achieved work generally focuses on aerial video with moderately-sized objects based on feature extraction. However, the moving vehicles in satellite video imagery range from just a few pixels to dozens of pixels and exhibit low contrast with respect to the background, which makes it hard to get available appearance or shape information. In this paper, we look into the problem of moving vehicle detection in satellite imagery. To the best of our knowledge, it is the first time to deal with moving vehicle detection from satellite videos. Our approach consists of two stages: first, through foreground motion segmentation and trajectory accumulation, the scene motion heat map is dynamically built. Following this, a novel saliency based background model which intensifies moving objects is presented to segment the vehicles in the hot regions. Qualitative and quantitative experiments on sequence from a recent Skybox satellite video dataset demonstrates that our approach achieves a high detection rate and low false alarm simultaneously.

## 1. Introduction

An increasing number of commercial earth observation satellites have been launched over the past decades that generate large quantities of satellite imagery with resolution of 1 m (Ikonos) or even better (QuickBird, geoEye), and most related satellite imagery based works experiment on their imagery. In addition, the SkySat-1 satellite launched by Skybox Imaging is the first civilian satellite to film a target in panchromatic video for up to 90 s. This real time satellite imagery empowers global business, including oil storage monitoring, disaster response [1], ecosystem disturbance detection [2] and maritime monitoring [3,4], etc. The main data source of today’s intelligent transportation system is generally from ground-fixed or aerial-based cameras or sensors. The major drawback of these kinds of imagery is the limited spatial coverage. As a result, researchers start to put attention to higher space. With great potential and advantage in the field of wide area monitoring, satellite video has become a new powerful way for traffic management.

As the foundation of intelligent traffic guidance and military and homeland surveillance, vehicle detection has been well-studied but is still a valuable research field. The existing vehicle detection algorithm is mostly designed for ground-sampled images or aerial images. There is also some achieved work focusing on vehicle detection in still satellite images, and they deliver good results while the same work in satellite videos has been scarcely considered. Therefore, a practical approach designed for small moving vehicle detection in consecutive satellite videos is required urgently to unlock the full potential of earth observed satellite videos.

**Figure 1:**
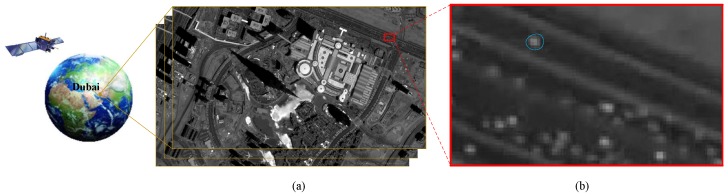
Skybox satellite video sequences. (**a**) is a frame of a skybox satellite video sequences. (**b**) is the enlarged part of the red labelled area in (**a**). The vehicle covers only several pixels in the image.

As a new point of access to earth observation, the space-based surveillance platform brings several challenges for real-time video analysis. The field view of Figure 1, is approximately 1.8 × 1.1 ${\mathrm{km}}^{2}$. Our statistics show that there are over one-hundred vehicles in Figure 1a. Compared to aerial images, vehicles in satellite videos range from only a few pixels to a dozen pixels. The small vehicles in satellite video sequences also exhibit low contrast to the background. Consequently, no available appearance or shape information can be extracted to carry out a common classification algorithm. Another difficulty for the vehicle detection lies in the much more complicated background of the satellite videos. High spatial coverage leads to large field of view and complete road information but messy scenes and lots of obstacles simultaneously. Besides the common objects in aerial image, like varying magnitudes of buildings and trees, the slowly moving airplanes can also be found in the satellite video sequences. Accounting for the characteristics of the satellite videos mentioned above, the traditional vehicle detection algorithm can not perform well for low detection accuracy. The challenge is detecting moving vehicles accurately while suppressing the false alarms induced by the same-size obstacles.

The vehicle detection approaches in aerial imagery have been well studied in recent research work [5,6,7,8,9,10]. In aerial images, the resolution is high enough to utilize the shape or appearance models of vehicles to satisfy the demand for detection. Even the component-based vehicle detection approach can be applied for the aerial imagery [10]. However, satellite video sequences cannot provide the detailed information of vehicles because of the limited resolution. Though less appearance information of vehicles can be utilized for detection, some methods are still proposed for object detection in high-resolution satellite imagery. Larsen et al. [11] detect vehicles by using an elliptical blob detection strategy and separating vehicles from non-vehicular objects by using a k-NearestNeighbor (KNN) classifier with various classical features. Sharma et al. [12] proposed the approach by using Bayesian background transformation (BBT) to detect change between the current image and the estimated background, but it is hard to obtain the proper background. Jin et al. [13] use a morphological shared-weight neural network (MSNN) to detect vehicles in Ikonos imagery and Zheng et al. [14] propose an artificial immune approach to detect vehicles by learning a set of templates for vehicle detection in QuickBird (QB) imagery. In [15], vehicles are detected by using adaptive boosting in combination with Haar-like features, and vehicle queues are detected using line extraction techniques. A blob detection algorithm for vehicle detection was adopted by Hinz and Stilla [16]. In [17], Pesaresi et al. located vehicles by manually selecting the target points, and then using morphology methods to refine the location of the vehicles. Some of the mentioned methods use the blob detection algorithm for vehicle detection. However, there are a lot of objects appearing similarly to vehicles in satellite video sequences, which might lead to confusion. Another approach mentioned above uses the road information obtained by geographic information system (GIS), or edge detection to segment roads from other parts of the scene. However, they always use prior knowledge of the threshold value for segmenting roads that cannot be extensively used in different images.

In this work, the motion heat map and local saliency map based small moving vehicle detection approaches are presented. For the satellite video sequence, the resolution is limited so that common shape or appearance based methods for object detection no longer make sense. Therefore, we choose the background subtraction to deal with the real-time segmentation of objects in our work. Illumination change, motion parallax caused by 3D structure and complicated background often lead to false detections in the background model. Different from single satellite image, satellite video sequence can provide spatio-temporal information. Therefore, kinematic information is fully exploited. To suppress the false alarms, we build a motion heat map in the first step of our vehicle detection. The motion heat map reflects the possible motion regions in the image, which would limit the sphere of detection for vehicles. Traditional background modeling using grayscale images delivers a low detection rate when facing a complicated background. In our work, we enhance the grayscale images by applying the local salience detection. A saliency based Gaussian mixture background model is proposed for real-time detection and segmentation. We conduct experiments on the satellite video sequence using the proposed method, and results show a high detection rate with a low false alarm rate.

This paper is organized as follows: the processing framework is described in Section 2. Experiments and results are presented in Section 3, and we conclude in Section 4.

## 2. The Proposed Framework

**Figure 2:**
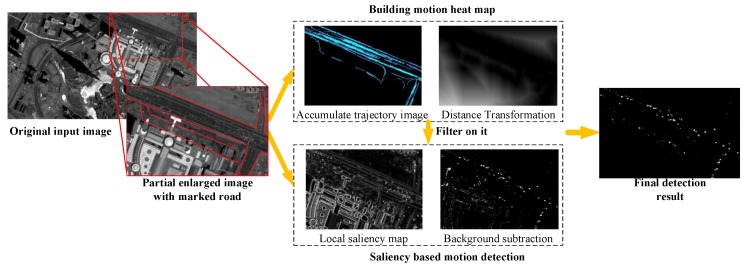
The proposed framework. The partial original satellite image is enlarged with red lines as marked roads. The proposed method contains two steps: building motion heat map and saliency based motion detection. In addition, the main process results of the two steps are displayed.

To reliably detect moving vehicles with maximal detection rate and minimal false positives, we propose a two-step approach: (i) build motion heat map, and (ii) local saliency based background model. The overall algorithm framework is shown in Figure 2.

### 2.1. Building Motion Heat Map

Traditional moving object detection methods including frame difference and background subtraction are applied to the whole image. Some instability factors such as illumination change, different weather and sway of branches in the wind make the background more complicated and messy. If the illumination changes, the grayscale of background pixels will change as well. This makes it sensitive to weak illumination change. The background in our experiment image is complicated and messy. There are also some moving shadows that have arisen from planes or buildings due to the sunlight. The buildings and trees result in the motion parallax in the motion detection. Simply applying the traditional detection method delivers low detection rate and high false alarm. In urban areas, however, moving vehicles are driven on the fixed regions, including different highroads and narrow pathes. If the regions where motion would take place have been already segmented in advance, it would be effective to suppress the false detections. In [15] , ROIs (regions of interest) are obtained by using the road data of GIS, but it is difficult to align the image to the road data precisely and automatically. In order to obtain higher detection accuracy with suppression of the false alarm, we propose to build motion heat map before detection of moving vehicles.

#### 2.1.1. Motion Detection

Firstly, background modeling is applied to detect moving objects. In addition, motion detection is performed by a classical ViBe (Visual Background extractor) [18] method, which shortens the modeling time and performs well when it comes with background changing abruptly. Accounting for the relative motion between the satellite and the earth, background motion exists in the satellite video sequences; however, the background motion is so slow that we need not do the motion compensation, and ViBe is also robust to the slow background motion.

The main principle of ViBe algorithm is as follows: let p(x) denote the value of the pixel, *x*, and use a set of sample values M(x)={p1,p2,...,pN}(N=20,empirically) as a pixel model. To classify a value p(x) as a background or foreground pixel value, a sphere $S_{R}\left(p\left(x\right)\right)$ of radius R centered on p(x) is defined, and the cardinality of the set intersection of $S_{R}\left(p\left(x\right)\right)$ and the set of samples M(x) is calculated. If the cardinality is higher than the threshold $T_{min}$, the pixel *x* is classified as a background pixel. To initialize the model, for a pixel in a frame, randomly select the value of neighbor pixels as its sample values. To adapt to the change of scene, the model is updated regularly. When a pixel is classified as background pixel, it has the probability of a certain one to update its background model by selecting the value of a sample pixel in its set of samples to fill the update. In addition, when the foreground has been classified as the background for K times, then update it as the background.

#### 2.1.2. Trajectory Based False Alarm Filter

After background modeling in the last step, we get a preliminary motion detection result for each frame, which contains not only the partial true moving objects, but also the bothersome false detections. For each connected domain, we suppose it to be an isolated detection. In some cases, false detections can be rejected based on their size; however, here we are attempting to detect super-small objects whose size is on the same order as noise. For this reason, trajectory information is utilized to distinguish the real targets from false ones. In order to build the false-alarm filter, the following steps are implemented.

Given the frame-by-frame detections, a set of trajectories are extracted from the detections by employing a Hungarian algorithm. The Hungarian algorithm is applied as follows: assuming there are *m* targets detected on the $t_{th}$ frame, and *n* targets detected on the ${\left(t+1\right)}_{th}$ frame, the targets on two frames have one-to-one correspondence. Let $C_{ij}$ denote the Euclidean distance between the $i_{th}$ target in the $t_{th}$ frame and $j_{th}$ target in the ${\left(t+1\right)}_{th}$ frame. $X_{ij}$ denotes the association relationship between the $i_{th}$ target in the $t_{th}$ frame and $j_{th}$ target in the ${\left(t+1\right)}_{th}$ frame. If the $i_{th}$ target in the $t_{th}$ frame and $j_{th}$ target in the ${\left(t+1\right)}_{th}$ frame turns out to be the same target, then the value of $X_{ij}$ equals 1; otherwise, it would equal 0. Targets have one-to-one correspondence between adjacent frames. As with Equation (1), the final solution should minimize the total distance. The association problem can be described as follows:

(1) $$z=\min\sum_{i=1}^{m}\sum_{j=1}^{n}C_{ij}X_{ij},$$

(2) $$\sum_{i=1}^{m}X_{ij}=1,j=1,...,n,$$

(3) $$\sum_{j=1}^{n}X_{ij}=1,i=1,...,m,$$

(4) $$X_{ij}\in \left\{0,1\right\}.$$

Accounting for the existence of false detections, the quantity of detections is not the same between adjacent frames, if m<n, the number of detected targets in the ${\left(t+1\right)}_{th}$ frame is more than it is in the $t_{th}$ frame, then the targets that have not been associated would be regarded as new targets; otherwise, if *m* ≥ *n*, then retain the targets which are not associated for two frames, and judge them as a false alarm or real detection by the following association result.

For the real moving vehicles, the trajectories should be continuous and straight-line in the temporal domain. Hence, we retain the trajectories whose length is longer than the threshold $T_{length}$ to guarantee the trajectory is generated by vehicle motion and the trajectory direction should remain stable in every *w* frames.

**Figure 3:**
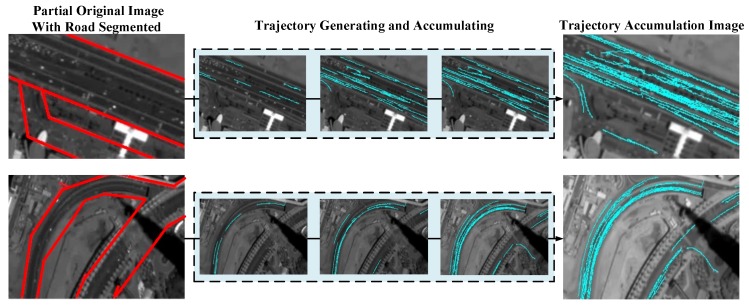
The process of trajectory generation and accumulation. Two roads of the original image are segmented. The trajectories are generating and accumulating with time, the final trajectory accumulation image is displayed on the right, and the trajectories have occupied the road area.

As shown in Figure 3, trajectories are generated and accumulated with time. In addition, the trajectories would occupy all the roads with enough accumulation. In this paper, 900 frames of the satellite sequence have been utilized for accumulating the trajectories to better reveal the road location.

#### 2.1.3. Motion Heat Map

The retained trajectories have high credibility belonging to the moving objects. To build the motion heat map, distance transformation is applied. Distance transformation is a special kind of transformation on the binary image, which results in a grayscale image. It assigns the value of the pixel *m* according to the Euclidean distance between *m* and the pixel *n*, which is the nearest pixel to *m* belonging to the trajectories. In addition, the distance transformation image is calculated as below:

(5) $$\mathrm{dis}\left(m,n\right)=\sqrt{{\left(x_{m}-x_{n}\right)}^{2}+{\left(y_{m}-y_{n}\right)}^{2}},$$

(6) $$I_{dt}\left(m\right)=\min_{n\in I_{trajectories}}\left(\mathrm{dis}\left(m,n\right)\right).$$

The result of distance transformation on the trajectory image reflects the probability of motion happening. High value means the pixel is far from the motion regions. Thus, the regions are rejected if the value of them are higher than the threshold $T_{heat}$. Then, the motion “hot” regions are segmented. For segmentation of motion “hot” region, the threshold $T_{heat}$ should be proper, if we set it too high, motion regions could not be segmented completely; if too low, the area that contains trees or buildings would be regarded as motion regions, which results in more false alarms. In this paper, $T_{heat}$ is set to 15 of the pixel value, as an empirical value.

**Figure 4:**
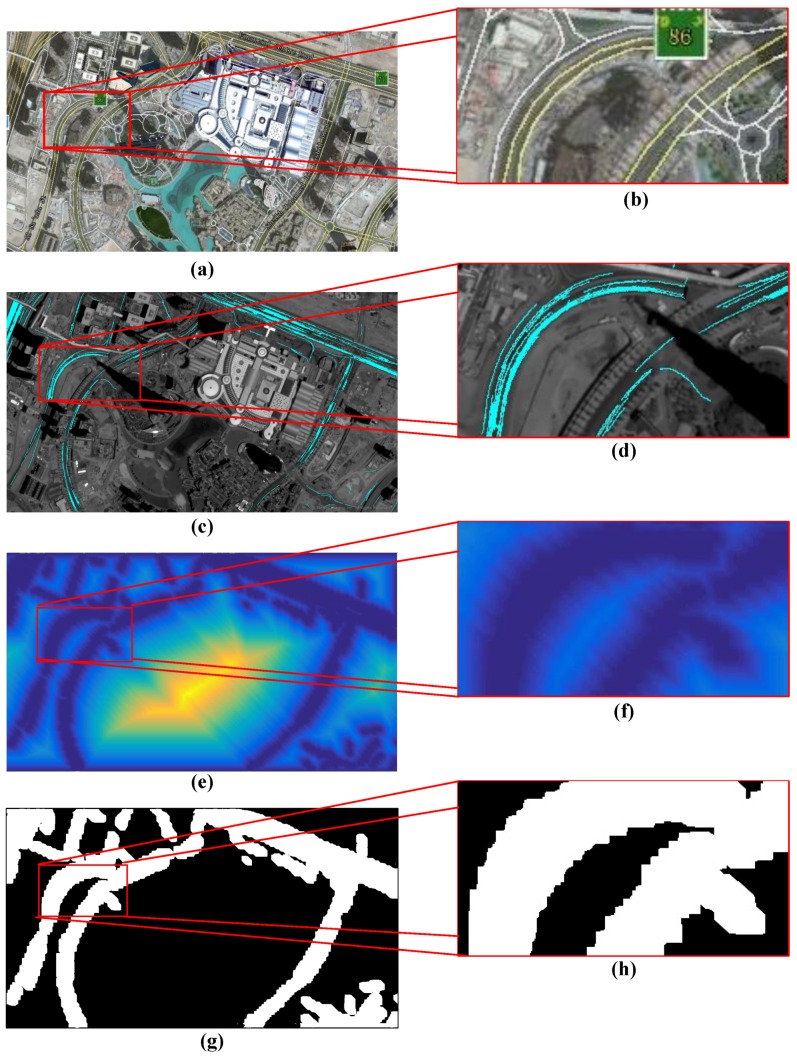
The main process result of building motion heat map. (**a**): real scenario captured by Google Earth; (**b**): partially enlarged image of (**a**); (**c**): accumulated trajectory image; (**d**): the partially enlarged image of (**b**); (**e**): the motion heat map; (**f**): the partially enlarged image of (**e**); (**g**) segmented result of motion heat map; and (**h**): the partially enlarged image of (**g**).

As shown in Figure 4, the motion heat map generates from the trajectory image. For better exhibition, we capture the real scenario by Google Earth (Google, Santa Clara, CA, USA), with roads segmented by yellow lines and the grayscale motion heat map transformed to an energy map. If the region has more energy, it means it is farther from the motion region. From (e) and (f) in Figure 4, we could observe that the road regions show low energy and the energy is larger farther from the road. In addition, from the comparison of (a) and (g) in Figure 4, we can reach the conclusion that the road regions are mainly segmented.

### 2.2. Local Saliency Map Background Model

Given the motion heat map we obtained in Section 2.1, the following work we do is only limited to the motion “hot” region, which means the most possible place motion would take place.

In this section, we propose a novel background model based on a saliency map to detect multiple small moving vehicles in the satellite video sequence. As a typical method for motion detection, background subtraction has its remarkable advantages, including the good adaptation to a complex background and accurate detection performance. However, a low contrast of vehicles to the road makes it difficult to segment objects clearly from the background only by traditional methods. In order to achieve better performance, we deviate from the grayscale image based object detection method and propose a novel background model based on local saliency map.

#### 2.2.1. Building Local Saliency Map

The first step of our detection is the local saliency detection. Most motion detection approaches perform better when the targets have good spatial or temporal saliency, which means the targets are compact in spatial distribution in a scene or have prominent actions in video sequences [19]. On accounting for that it seems more attractive of vehicles in the local region than the global image in the satellite video sequences, local saliency is employed to intensify the objects as the first step of background model.

Several saliency detection algorithms, such as [19,20,21,22], are proposed in recent research works. In this work, the saliency detection method similar to [19] is adopted to finish local saliency detection. Compared to the algorithm proposed in [20,21,22], the algorithm proposed in [19] performs better in local saliency detection when it comes with dim contour information. We compute local saliency over a sliding window of images. Let the value of pixel *i* of the image be denoted as $I_{i}$, and let *ω* denote the region of the local window occupied in the image, and $∥\cdot ∥$ represent the grayscale distance metric. Then, the local saliency value of a pixel *k* in an image is defined as

(7) $$Sal\left(k\right)=\sum_{\forall i\in \omega }∥I_{k}-I_{i}∥.$$

In this equation, $I_{i}$ is in the range of [0, 255], which can be expanded as:

(8) $$Sal\left(k\right)=∥I_{k}-I_{1}∥+∥I_{k}-I_{2}∥+\ldots +∥I_{k}-I_{N}∥,$$

where *N* is the total number of pixels in the sliding window, which is centralized by pixel *k*. According to the Equations (7) and (8), the size of the window could affect the result of the local saliency map. If the size is set too high, only the obvious and big objects are intensified, if too low, more noise will be generated. For the purpose of intensifying the moving vehicles in the image, the size of the window is set to 3 pixels, which is an empirical value.

**Figure 5:**
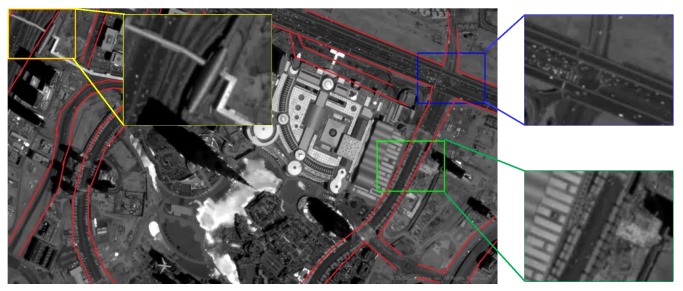
The original image with road boundary marked in red and three regions in bounding boxes are chosen to explain our method.

**Figure 6:**
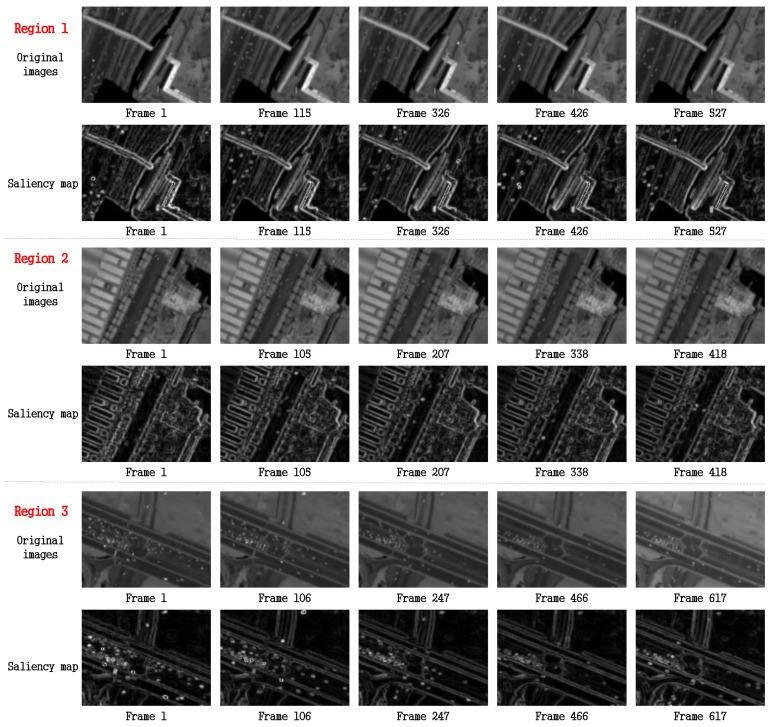
The comparison between grayscale original image and saliency map of the same region. Three regions are selected, and for each regions, two rows are displayed. For each regions, the first rows show the original image sequence of the region and the second rows show the saliency map of the region.

As shown in Figure 5, to better explain the effectiveness of our method, three regions of satellite images are selected. As we can see in Figure 6, the super small moving objects are highlighted in the local saliency map. Some vehicles on the road are so faint that we would ignore them easily, but they are much more recognizable and attractive in the saliency map compared to the original grayscale image.

For each pixel *k* in an input grayscale image, we compute its local saliency value Sal(k), and then we acquire a saliency map. After this step, the vehicles would show higher contrast to background compared with grayscale image.

#### 2.2.2. Local Saliency Based Motion Detection

In this section, the ViBe background model [18] proposed by Barnich is applied again to finish the background subtraction, and the principle of ViBe has been explained in Section 2.1.1. As we know, ViBe is a very outstanding method for detection, but the characteristic of satellite image makes the vehicles show low contrast to roads, which results in lower detection rate. Different from the traditional background subtraction on the grayscale image, we apply the ViBe on the local saliency map, which we obtain from the previous step. The local saliency map makes the background subtraction detection more sensitive to the vehicles in the satellite image.

**Figure 7:**
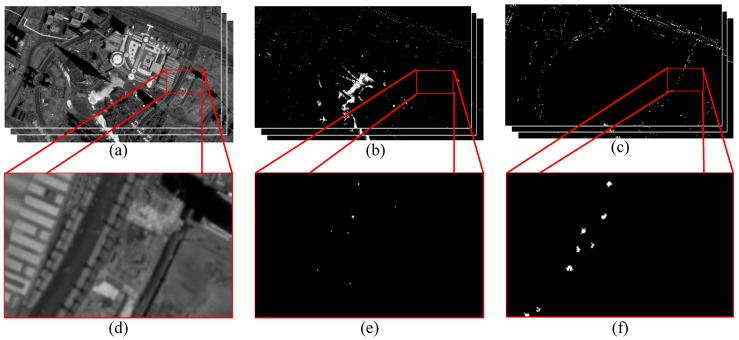
(**a**): the original image; (**b**): the detection result of ViBe (Visual Background extractor); (**c**): the detection result of our method; (**d**): partially enlarged image of the original image; (**e**): the partially enlarged image of (**b**); and (**f**): the partially enlarged image of (**c**).

Figure 7 shows the comparison of the original ViBe result and our method. To better exhibit the result, we select a road region and enlarge part of the region to show the results of the two algorithms, respectively. Figure 7e is the enlarged result of the original ViBe, although several obvious vehicles are detected, and the vehicles that show low contrast to roads cannot be detected. In addition, Figure 7f is the enlarged result of our method, and the result is very obvious—almost all of the vehicles on the road are detected, and the detected vehicles are segmented clearly.

## 3. Experiments

We conduct the experiment to evaluate the performance of the proposed approach. In this section, we will describe the details of the experiment. In addition, the reference parameters used in this paper are presented as follows. The $T_{length}$ and the *w* in Section 2.1.2 are set to 40 pixels and 15 pixels, respectively. The $T_{heat}$ in Section 2.1.3 is set to 15 of the pixel value. The size of the window in Equation (6) is set to 3 pixels.

### 3.1. Dataset

We validate our approach on a dataset of a satellite video sequence. The video sequence is collected by SkySat-1 on 9 April 2014 from Burj Khalifa in Dubai, which is the tallest building in the world. The video sequences contain 900 frames, with a footprint of approximately 1.8×1.1 ${\mathrm{km}}^{2}$. The resolution is 1.5 m and the frame size is 1280×720 [23] pixels at 30 frames per second. There are hundreds of objects in each frame, and each object covers roughly 5×5 pixels. It is a challenging work to detect vehicles in such a scene, not only because of the super-small size of the target but also the change of illumination and shadow. For quantitative evaluation of our approach, we select four subsequences from the dataset. Each subsequence contains five frames. The first five frames are frame 20 to frame 24, which is the stable stage of dark global illumination. Frame 885 to frame 889 is the second group with bright global illumination. The other two groups are frame 676 to frame 680 and frame 706 to frame 710, which are corresponding to the illumination change stage and the shadow change stage. For verification of our method, we manually label the ground truth. The labelled result contains the area moving vehicles occupy in the satellite video sequences.

### 3.2. Evaluation Metrics

We identify the detection results as follows: for each connected domain in the motion detection result, the one which has at least one pixel overlap with ground truth is supposed to be a true positive (TP); otherwise, it is regarded as a false positive (TP), and false negative (FP) is the moving vehicle which has not been found.

### 3.3. Qualitative Evaluation

For qualitative evaluation of our method, we conduct two experiments.

**Figure 8:**
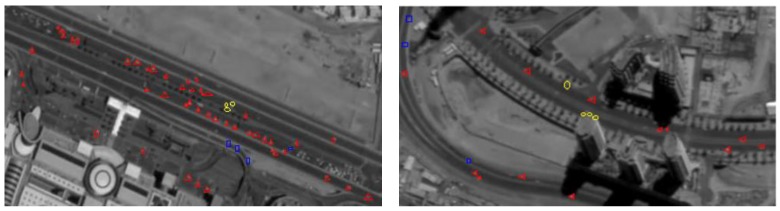
Final detection results. Truth Positive (TP) is labelled by red triangles. False positive (FP) is labelled by yellow ellipses. False Negative (FN) is labelled by blue squares.

In the first experiment, we analyse our detection results. Two regions that contain roads are selected and enlarged for better observation. As we can see in Figure 8, the true positives (TP) are labelled by red triangles. There are several true moving vehicles labelled by blue squares, which means they have not been detected. The reason why they are rejected is not the same. One main reason is that some vehicles are almost still in the video sequences for the low speed. Another reason is that the gray value of vehicles shows high similarity to the background. False Positive (FP) is labelled by yellow ellipse which means wrong objects are regarded as moving vehicles for the illumination change and the parallax caused by high buildings. Although we have built the motion heat map to decrease the false alarms made by parallax, the edges of buildings which are very close to the road are also classified to the motion “hot” regions, which would cause the inevitable false alarms. However, most of the moving vehicles have been detected. The experiments we conducted show that our method is a practical solution for small moving vehicle detection in satellite video sequences.

**Figure 9:**
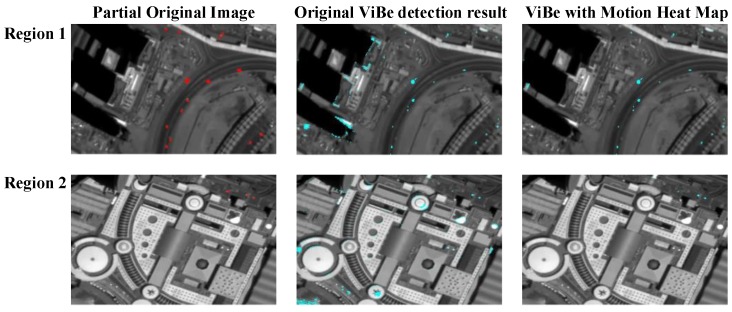
Comparision result of original ViBe(Visual Background extractor) detection and ViBe with motion heat map. In every row of the figure, from left to right: the original image with manually marked ground truth in red, the original ViBe detection result, and ViBe detection result with motion heat map.

In the second experiment, for better illustration of the effectiveness of the proposed motion heat map, we take the original grayscale video sequence as the input sequence and generate two different results with or without motion heat map. As we can see in Figure 9, two regions that contain buildings are selected from the original image. The original ViBe detection result contains many false alarms on the edges of buildings. However, in our detection result with motion heat map, false alarms are significantly reduced while the true moving vehicles are retained on the result image.

**Figure 10:**
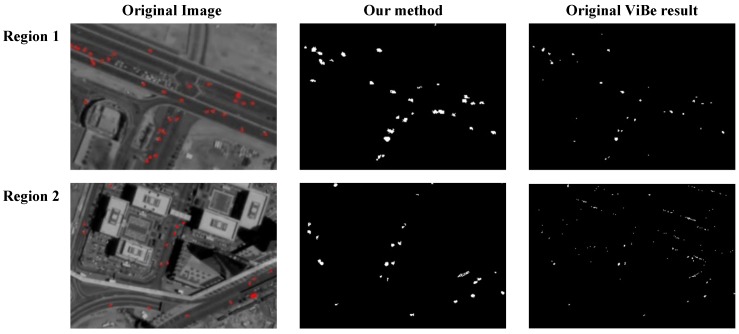
The comparison between grayscale image background and saliency based background. We select two regions in the original image. In each row of the figure, from left to right: the original image with manually marked ground truth in red, the saliency based detection result, and the grayscale image background result.

To discuss the effectiveness of the proposed local saliency map, we compare our local saliency based detection result with a ViBe result. As shown in Figure 10, the original image is dark and shows low contrast, and the detection result using the original ViBe is not prominent, and the detected moving vehicles only occupy a few pixels with many false alarms on the edge of buildings. In contrast, the result of the proposed method performs much better, and almost all moving vehicles are segmented clearly with few false detections appearing in the detection results. The result indicates the effectiveness of the proposed local saliency map and motion heat map.

### 3.4. Quantitative Evaluation Experiment

#### 3.4.1. Quantitative Evaluation Metrics

For quantitative evaluation, we use standard evaluation measures: true positives (TP), false positives (FP), false negatives (FN), percision and recall and *F*-score. *F*-score (F1-score) is the harmonic mean of percision and recall. percision, recall and *F*-score are calculated as:

(9) $$precision=\frac{TP}{TP+FP},$$

(10) $$recall=\frac{TP}{TP+FN},$$

(11) $$F-score=2*\frac{precision}{precision+recall}.$$

#### 3.4.2. Quantitative Evaluation

To illustrate better about the performance of our method, we conduct two comparative experiments. For better exhibition effect, the best performance result is labelled in red font and the worst performance result is labelled in blue font in the next two tables.

In the first experiment, we take the original grayscale image as the input imaginary and compare our detection method with the original ViBe method. As shown in Table 1, we use TP, FP and *F*-score to evaluate the detection result from the two methods. The proposed method performs better with keeping higher TP and reducing the FP in different situations compared with the original ViBe. In addition, the *F*-score also keeps a stable state in the proposed method. Due to the images showing dark illumination during 20–24 frames and 676–680 frames, the grayscale background subtraction has poor performance with low TP, and it performs better when the images show high contrast during 320–324 frames. While our saliency background subtraction shows stable and better performance during the whole detection process, the dark or bright illumination has little influence on our method. This is due to the employment of motion heat map and local saliency map. Motion heat map guarantees the detection activity limited to the valid regions instead of the impossible regions such as buildings and trees. In addition, the next local saliency map intensifies the contrast between vehicles and background.

**Figure 11:**
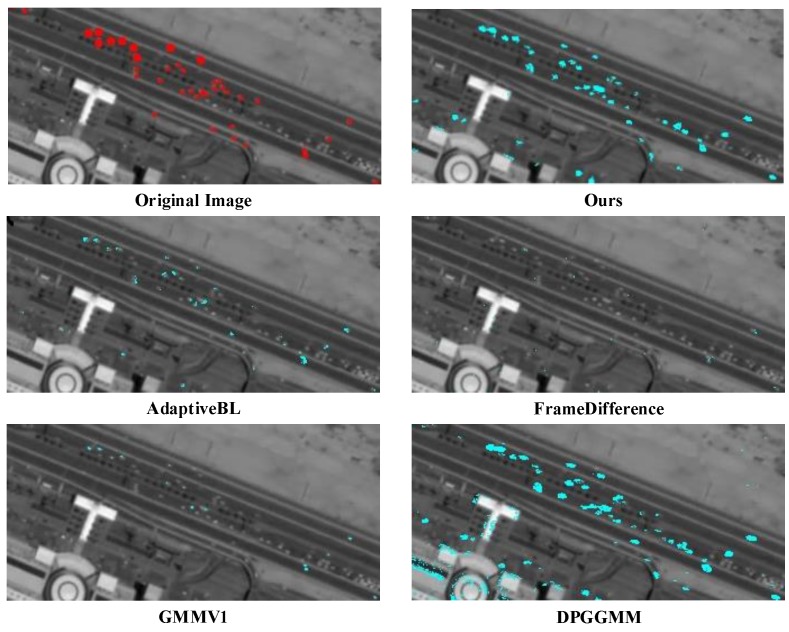
Partially enlarged details of detection results using different methods.

In the second experiment, five typical detection methods including an improved adaptive Gaussian mixture modeling (GMMV1) proposed in [24], Adaptive Background Learning (AdaptiveBL) [25], Frame Difference (FrameDiff) and DPGrimson Gaussian Mixture Modelling Background Substraction (DPGGMM) [26] from open source library (BGSLibrary) [27] are chosen. In addition, the classical ViBe [18] method can also be chosen to compare with our proposed method. We replace our saliency background model by these models one by one. Table 2 shows the comparative results. We can find that AdaptiveBL, ViBe and DPGGMM show higher recall than other methods but lower precision which are beyond exception. The recall of GMMV1 and FrameDiff is too low to be a good choice. Our method performs much better than other methods, which shows much higher recall and precision. In addition, the *F*-score of our method is the highest, which means the best performance. In addition, the detection results of the above methods are shown in Figure 11. With less false alarms and more correct detections, our method is much more suitable for moving vehicle detection on the satellite imagery. Dynamic detection results of our method are presented in the appendix demo.

## 4. Conclusions

Satellite video has wide application prospects in surveillance for ground vehicles over a wide area. In this paper, we focus on the detection of small moving vehicles in high resolution satellite video sequences in this paper. The saliency background model is proposed to intensify the moving objects and improve the accuracy of object detection. The detection refinement is based on the motion heat map. Low resolution and low contrast between the vehicles and its surroundings in satellite imagery make it hard to have good performance to detect moving vehicles by traditional methods. The presented approach can accurately detect hundreds of small objects in the satellite image with low false-alarm rate.

The Skybox satellite imagery can provide excellent information on traffic in cities. In addition, we experiment with our method in the Skybox satellite video sequence, and we compare the proposed method with a traditional detection approach. The experimental results show that our method has better performance with higher detection rates and low false-alarm rate.

The approach in this paper provides a new idea for small object detection and thus helps to unlock the full potential of a large amount of satellite images. Moreover, real-time detection and tracking of the small moving vehicles is also a challenging part for us in the future work. We will investigate more about how to enhance the detection rate of small vehicles in satellite imagery.

## Figures and Tables

**Table 1:** The detection result of our method and original ViBe (Visual Background extractor) on different subsequences.

FrameID		20–24	320–324	676–680	885–889	Total
Ground truth (GT)		1243	777	802	761	3583
TP	ViBe	986	475	524	693	2678
	Our method	1093	528	594	627	2842
FP	ViBe	5191	5374	4742	6310	21617
	Our method	1494	709	426	811	3440
F1	ViBe	0.2657	0.1469	0.1734	0.1712	0.1938
	Our method	0.5707	0.5698	0.6611	0.5702	0.5929

**Table 2:** The detection results of different methods.

Method	Ours			ViBe			AdaptiveBL		
**Index**	**Recall**	**Precision**	**F1**	**Recall**	**Precision**	**F1**	**Recall**	**Precision**	**F1**
20–24	0.88	0.35	57.07%	0.79	0.12	26.57%	0.98	0.03	5.00%
320–324	0.86	0.34	56.98%	0.77	0.06	14.69%	0.97	0.03	5.63%
676–680	0.76	0.35	66.11%	0.67	0.06	17.34%	0.58	0.13	36.36%
885–889	0.82	0.33	57.02%	0.88	0.08	17.12%	0.73	0.01	3.35%
**Average**	0.82	0.35	59.29%	0.78	0.08	19.38%	0.81	0.05	12.58%
**Method**	**GMMV1**			**DPGGMM**			**Framediff**		
**Index**	**Recall**	**Precision**	**F1**	**Recall**	**Precision**	**F1**	**Recall**	**Precision**	**F1**
20–24	0.26	0.03	21.34%	0.98	0.03	5.74%	0.43	0.03	12.72%
320–324	0.26	0.06	36.74%	0.92	0.02	4.85%	0.33	0.04	22.58%
676–680	0.09	0.01	16.57%	0.88	0.03	6.72%	0.32	0.03	16.48%
885–889	0.43	0.16	54.47%	0.90	0.03	5.92%	0.32	0.03	16.78%
**Average**	0.26	0.05	32.28%	0.92	0.03	5.81%	0.33	0.03	15.63%

## Supplementary Materials

The following are available online at http://www.mdpi.com/1424-8220/16/9/1528/s1, Video S1: Small Moving Vehicles Detection in Satellite Video.
